# The Influence of Genetic Polymorphisms on the Expression of Interleukin-1beta, Prostaglandin E2 and Tumor Necrosis Factor Alpha in Peri-Implant Crevicular Fluid: A Cross-Sectional Study

**DOI:** 10.3390/ijms25010651

**Published:** 2024-01-04

**Authors:** José Maria Cardoso, Ana Clara Ribeiro, João Botelho, Luís Proença, Susana Noronha, Ricardo Castro Alves

**Affiliations:** 1 Egas Moniz Center for Interdisciplinary Research (CiiEM), Egas Moniz School of Health & Science, Campus Universitário, Quinta da Granja, 2829-511 Almada, Portugal; acribeiro@egasmoniz.edu.pt (A.C.R.); jbotelho@egasmoniz.edu.pt (J.B.); lproenca@egasmoniz.edu.pt (L.P.); ralves@egasmoniz.edu.pt (R.C.A.); 2 Periodontology Department, Egas Moniz School of Health & Science, Campus Universitário, Quinta da Granja, 2829-511 Almada, Portugal; 3 Molecular Biology Laboratory, Egas Moniz School of Health & Science, Campus Universitário, Quinta da Granja, 2829-511 Almada, Portugal; 4 Periodontology Department, Faculdade de Medicina Dentária da Universidade de Lisboa, Cidade Universitária, R. Prof. Teresa Ambrósio, 1600-277 Lisboa, Portugal; susanacnoronha@gmail.com

**Keywords:** dental implants, genotype, interleukin-1, interleukin-1alpha, peri-implantitis, polymorphisms

## Abstract

The aim of this study was to evaluate the possible relationships between polymorphisms in the interleukin-1 (*IL-1*) *A*, *IL-1B*, and *IL-1RN* genes and concentrations of the inflammatory mediators IL-1β, tumor necrosis factor-alpha (TNF-α), and prostaglandin E2 (PGE2) in peri-implant crevicular fluid (PICF). A cross-sectional analytical study was conducted on 51 patients with dental implants. Samples from the buccal mucosa were obtained, and genetic analysis was performed using the real-time polymerase chain reaction (PCR) technique for *IL-1A* and *IL-1B* and PCR and restriction fragment length polymorphism analysis for *IL-1RN*. For the biochemical analysis, the concentrations of IL-1β and TNF-α were analyzed using multiplexed fluorescent sphere immunoassays, and PGE2 by enzyme-linked immunosorbent assay. In patients with detected *IL-1RN* polymorphism, there was an increase in the concentration of the three mediators with statistically significant differences in the mean values of TNF-α and PGE2, regardless of peri-implant health status (*p* = 0.002 and *p* = 0.049, respectively). The concentrations of all three mediators were positively and significantly correlated (IL-1β vs. TNF-α Rho = 0.480, *p* < 0.001; IL-1β vs. PGE2 Rho = 0.382, *p* = 0.006; and TNF-α vs. PGE2 Rho = 0.528, *p* < 0.001). We can conclude that the *IL-1RN* polymorphism exerts an influence on the PICF immune response, which may explain the influence of this genetic polymorphism on the occurrence of peri-implantitis.

## 1. Introduction

Although endosseous implants demonstrate significant success and survival rates, it is important to recognize that complete prevention of biological complications is not achievable [1,2,3]. One of the biological complications around dental implants is peri-implantitis, which is a plaque-associated pathological condition that occurs in the tissues around dental implants and is characterized by inflammation of the peri-implant mucosa and progressive loss of the supporting bone [4,5]. According to the new classification of periodontal and peri-implant diseases, in the absence of previous examination data, the diagnosis of peri-implantitis should be based on the combination of the presence of bleeding and/or suppuration on gentle probing, probing depths of ≥6 mm, and bone levels ≥3 mm apical to the most coronal portion of the intraosseous part of the implant [4]. Although specific bacteria, dental plaque, and environmental factors are associated with peri-implant disease, there is currently a lack of reliable indicators to predict the occurrence and severity of peri-implantitis [6]. Important risk factors/indicators have been identified, including a history of periodontitis, poor plaque control, and no regular supportive peri-implant care following implant therapy [7,8]. Less conclusive evidence was found for smoking and diabetes, or local factors such as the presence of submucosal cement following prosthetic restoration of the implant, or positioning of implants limiting access to oral hygiene and maintenance [7,8].

There is controversy regarding significant differences in the incidence of peri-implantitis and implant loss between smokers and non-smokers [7,8]. According to a review study on the influence of smoking on implant therapy, there is insufficient evidence on the effect of smoking on peri-implantitis, as well as on long-term implant loss [9]. On the other hand, in a study evaluating the phenomenon of corticalization of the peri-implant jawbone in tobacco smokers, a correlation was observed between smoking and changes in bone structure in radio textures near the implants [10]. Other studies have shown that smoking may affect the outcome of peri-implant disease treatment [11,12].

According to a recent systematic review of changes in molecular signatures associated with dental implant material in peri-implantitis, differences in transcriptomic signatures between peri-implant lesions and periodontal tissues may be related to titanium particles arising from dental implants [13]. In a comparative analysis of the transcriptome of periodontitis and peri-implantitis in humans, it was observed that although periodontitis and peri-implantitis shared a common genetic expression that was clearly distinct from healthy conditions, there were also unique genetic patterns that were differentially expressed only in peri-implantitis [14].

**Scheme 1:**
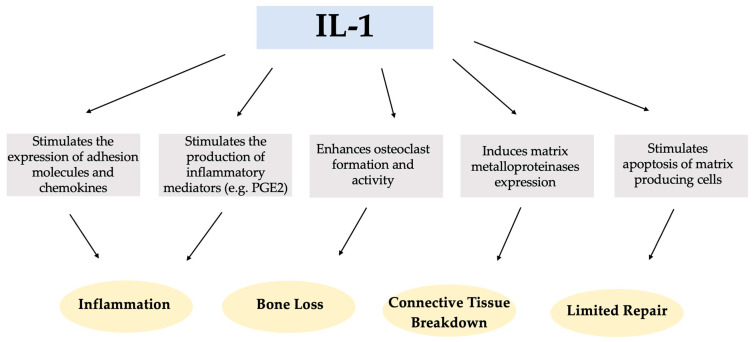
Potential impact of Interleukin-1 (IL-1) in the pathogenesis of inflammatory diseases.

Interleukin-1 (IL-1) is a multifunctional cytokine that plays a key role in the pathogenesis of inflammatory diseases, with a variety of diverse activities and functions in the immune response, inflammation, tissue breakdown, and tissue homeostasis, as shown in Scheme 1 [15,16]. 

The existence of two genetically and biochemically distinct IL-1 molecules, IL-1 alpha (IL-1α) and IL-1 beta (IL-1β), is well-established. These molecules exhibit comparable biological functions but share only 27% homology at the amino acid level [17]. Both forms of interleukin (IL1α and IL-1β) bind to a common receptor that is present at varying densities in numerous cell types. Within the IL-1 family of proteins, there is also the IL-1 receptor antagonist (IL-1Ra) [18]. IL-1Ra plays an anti-inflammatory role by preventing the transmission of pro-inflammatory signals and the immune response by using the same receptor as pro-inflammatory IL-1 [19]. 

Several studies have reported higher levels of IL-1β and tumor necrosis factor-alpha (TNF-α) in the peri-implant crevicular fluid (PICF) of implants with peri-implantitis than in healthy implants [20,21,22,23,24]. IL-1β, a powerful pro-inflammatory protein, plays a crucial role in the inflammatory process that occurs in the oral cavity. It acts as a key mediator in the production of prostaglandins, leukotrienes, and platelet-activating factors in various cell types. In addition, IL-1β stimulates osteoclast formation and contributes to bone resorption [25,26]. IL-1β is one of the candidate biomarkers that has been identified as being strongly associated with an increase in immuno-infiltrating cells in soft tissue samples obtained from peri-implantitis sites [27]. Another pro-inflammatory cytokine, TNF-α, shares several functions with IL-1β [28,29]. TNF-α also stimulates bone resorption and fibroblast apoptosis, resulting in limited repair of the periodontium, and increasing local secretion and activity of metalloproteinases that degrade connective tissue [30,31].

Prostaglandin E2 (PGE2) is a vasodilator that increases vascular permeability at sites of inflammation and also plays a role in bone resorption. Some studies agree that higher levels of PGE2 are found in the PICF of patients with peri-implant disease [20,32,33].

Genetic polymorphisms can influence gene expression, protein synthesis, and cytokine secretion [34]. Numerous observational studies have investigated the potential association between different gene polymorphisms and the development of peri-implantitis, with a significant emphasis on IL-1 [16,19,35,36,37,38,39]. Among them, the *IL-1A*, *IL-1B*, and *IL-1RN* genes have been studied. These genes encode IL-1α, IL-1β, and IL-1Ra, respectively [18,40]. The *IL-1A* (rs1800587) and *IL-1B* (rs1143634) genes are known to regulate the production of IL-1α and IL-1β, respectively [40,41]. They are located close to each other on the long arm of chromosome 2, specifically at position 2q13, and are considered potential candidates for genetic markers in peri-implantitis since the cytokines they encode play a crucial role in the development of the inflammatory response [19]. The presence of the *IL-1RN* allele 2 has been linked to a reduction in the production of IL-1Ra protein [18,42]. Furthermore, mononuclear cells obtained from individuals carrying the *IL-1RN* allele 2 exhibited an elevated production of IL-1β in vitro [43]. These findings indicate that the presence of the *IL-1RN* allele 2 can potentially disrupt the balance between IL-1β and IL-1Ra, leading to greater vulnerability or more pronounced symptoms of inflammatory diseases. Although there is no consensus in the literature regarding the role of genetic polymorphisms in the development of peri-implantitis, most studies have focused on *IL-1A* -889, *IL-1B* +3954, and *IL-1RN* variable number of tandem repeats (VNTR) polymorphisms. Some studies have found an association between the presence of these polymorphisms and peri-implantitis [16,19,36,37,44], while others have not [35,39,45,46].

Moreover, over the past 25 years, numerous single nucleotide polymorphisms have been associated with an increased incidence or severity of periodontal inflammation. The most studied polymorphisms were *IL-1A* -889 and *IL-1B* +3954. Meta-analyses seem to show an association between these polymorphisms and periodontal disease [47,48,49]. There is evidence that the presence of the composite positive genotype (i.e., the presence of at least one altered allele in the *IL-1A* -889 C/T and *IL-1B* +3954 C/T genes) increases susceptibility to periodontitis [47,50]. The sequence of immunopathological events in peri-implant infections has some similarities to periodontal infections [51]. 

Although there are studies that try to relate the existence of certain genetic polymorphisms and the presence of peri-implantitis, to our knowledge, there is only one study that relates genetic alterations with changes at the biochemical level in the PICF [35]. In this study, with 29 participants, the IL-1 polymorphism investigated had a minimal effect on the peri-implant crevicular immune response. A study with a larger sample size and better-defined criteria for cases of peri-implant health and peri-implantitis may help to evaluate the effect of these polymorphisms at the level of inflammatory mediators. The possible association between genetic polymorphisms and peri-implantitis must be based on an alteration in the individual’s immune-inflammatory response. Therefore, it is important that studies evaluating genetic polymorphisms in patients with peri-implantitis investigate the functional consequences of these polymorphisms. This can be obtained by analyzing the protein levels in PICF. Table 1 describes the various genetic polymorphisms that have been tried to be associated with peri-implantitis.

In the present study, we aimed to investigate the possible relationships between *IL-1A* -889, *IL-1B* +3954, and *IL-1RN* (VNTR) gene polymorphisms and the concentrations of the PICF mediators IL-1β, TNF-α, and PGE2. The main hypothesis of the study was that, in both healthy individuals and patients with peri-implantitis, an altered genotype of *IL-1A*, *IL-1B*, or *IL-1RN* would result in higher levels of pro-inflammatory mediators compared to patients without the altered genotype, and that this effect would be particularly pronounced in the peri-implantitis group. A second hypothesis was that the concentrations of the three mediators would be correlated in health and disease since they all orchestrate the same biological process. 

## 2. Results

### 2.1. Demographic and Clinical Characteristics of the Study Group

A total of 51 healthy individuals, consisting of 26 men and 25 women, with ages ranging from 38 to 90 years, were included in the study. The mean age in the peri-implant health group was 58.74 years and in the peri-implantitis group 59.17 years. The median values for age are 60 and 57.5 for the health and peri-implantitis groups, respectively. All the participants shared the same ethnic background (Caucasian) and were born in Portugal. Each participant had at least one dental implant in function. Cigarette smoking, history of periodontitis, and full-mouth plaque score were among the factors investigated for their prognostic significance. The details of the sample’s characteristics are shown in Table 2. Although there was a higher proportion of smokers in the disease group compared to patients with peri-implant health (54.2% vs. 33.3%, *p* = 0.134), the difference was not statistically significant. The peri-implantitis group demonstrated a higher prevalence of a positive history of periodontitis compared to patients with peri-implant health, albeit the differences were not significant (66.7% vs. 59.3%, *p* = 0.585). There was no statistical difference between the groups in terms of the full-mouth plaque score (FMPS). The implants were in function in the health group for 8.6 years and in the peri-implantitis group for 8.7 years. The mean time in function of the implants was not significantly different between the groups.

### 2.2. Genotype Frequencies and Genetic Models

The genotype frequencies obtained for the genes *IL-1A* -889, *IL-1B* +3954, and *IL1-RN* (VNTR), in the peri-implant health group, in the peri-implantitis group, and for the total sample are presented in Table 3.

With regard to the *IL-1A* -889 polymorphism, 59.3% of the subjects had the CC genotype, while 40.7% showed the CT or TT genotypes. In the peri-implantitis group, 54.2% had the CC genotype and 45.8% had the CT or TT genotypes. No statistically significant differences were found between the health and disease groups (*p* = 0.714).

With respect to the *IL-1B* +3954 polymorphism, in the healthy group, the genotype frequencies were 66.7% for the CC genotype and 33.3% for the CT and TT genotypes. For the group with diagnosed peri-implantitis, frequencies of 70.8% were obtained for the CC genotype and 29.2% for the CT and TT genotypes. No statistically significant differences were found in the proportions of the *IL-1B* gene polymorphism between the health and disease groups (*p* = 0.749). 

In relation to the *IL-1RN* polymorphism (VNTR), in the healthy group, the genotype frequencies were 85.2% for the 1/1 genotype and 14.8% for the altered genotypes (1/5 and 2/2). For the group with diagnosed peri-implantitis, the frequencies were 87.5% for the 1/1 genotype and 12.5% for less common genotypes. The distribution of the different genotypes for this polymorphism showed no differences between the groups in the proportions found (*p* = 0.811).

When the genotypes (homozygous/heterozygous variant) were analyzed separately for the *IL-1A* -889 and *IL-1B* +3954 polymorphisms it was not possible to perform the inferential analysis. When the genotypes were analyzed separately for the *IL-1RN* polymorphism (1/1, 1/5, and 2/2), inferential analysis was also not possible.

The odds ratio and corresponding 95% confidence interval are shown for the *IL-1A* -889 and *IL-1B* +3954 polymorphisms in the dominant and recessive genetic models in Table 4 and Table 5, respectively.

As shown in Table 4 and Table 5, the *IL-1A* -889 and *IL-1B* +3954 polymorphisms were not associated with the risk of peri-implantitis in the dominant and recessive models.

### 2.3. Influence of IL-1A -889, IL-1B +3954, and IL-1RN (VNTR) Polymorphisms on PICF Volume and Selected Biochemical Markers of the Peri-Implant Immune Response

The analysis of the influence of *IL-1A* -889, *IL-1B* +3954, and *IL-1RN* (VNTR) polymorphisms on the mean concentrations of IL-1β, TNF-α, and PGE2 (pg/μL) when the healthy and diseased groups were grouped, is described in Table 6.

For the *IL-1A* -889 polymorphism, it was observed that patients with the altered genotype exhibited higher mean concentrations of IL-1β (*p* = 0.518) and TNF-α (*p* = 0.313) and a lower mean concentration of PGE2 (*p* = 0.231) than patients without the presence of this polymorphism. However, these changes were not statistically significant. 

For the *IL-1B* +3954 polymorphism, it was observed that patients with the altered genotype had a higher mean concentration of TNF-α (*p* = 0.490) and lower mean concentrations of IL-1β (*p* = 0.776) and PGE2 (*p* = 0.543) than patients without genotype changes. However, these changes were not statistically significant. 

For the *IL-1RN* gene, it was noted that patients who had the polymorphism had higher mean concentrations of IL-1β (*p* = 0.339), TNF-α (*p* = 0.002), and PGE2 (*p* = 0.049) than patients who did not have this polymorphism. These differences were statistically significant (*p* < 0.05) for TNF-α and PGE2 levels. 

In patients with the IL-1 positive composite genotype, there was an increase in the mean TNF-α concentration (*p* = 0.364) and a decrease in the mean IL-1β and PGE2 concentrations (*p* = 0.713 and *p* = 0.634, respectively) compared to patients with the IL-1 negative composite genotype. However, these differences were not statistically significant (*p* > 0.05).

We also evaluated whether the influence of the genetic component at the biochemical level was more pronounced in individuals with peri-implantitis. For this purpose, only individuals with the IL-1 positive composite genotype or polymorphism in the *IL-1RN* (VNTR) were evaluated, comparing patients with peri-implant health and patients with peri-implantitis (Table 7).

It was observed that among patients with IL-1 positive composite genotype, patients with peri-implantitis had a higher mean volume of PICF (healthy 0.51 μL vs. peri-implantitis 0.66 μL, *p* = 0.042) and higher mean concentrations of IL-1β (healthy 15.31 pg/μL vs. peri-implantitis 23.65 pg/μL, *p* = 0.612) and PGE2 (healthy 119.06 pg/μL vs. peri-implantitis 186.54 pg/μL, *p* = 0.043) than healthy patients. These differences were statistically significant for PICF volume and mean PGE2 concentration. 

Regarding patients with the VNTR polymorphism, the mean volume of PICF and the mean concentration of PGE2 were higher in the disease group than in the healthy group. However, the differences in PICF volume and PGE2 concentration were not statistically significant (*p* = 0.660 and *p* = 0.464, respectively).

### 2.4. Correlation between Concentrations of Biochemical Markers (IL-1β, TNF-α, and PGE2)

IL-1β levels were positively and significantly correlated with TNF-α (rho = 0.480, *p* < 0.001) and PGE2 levels (rho = 0.382, *p* = 0.006). TNF-α levels were also positively related to PGE2 levels (rho = 0.528, *p* < 0.001) (Table 8). All three mediator concentrations were moderately positively correlated.

The data supporting the reported results are presented in Table S1 as supplementary material.

## 3. Discussion

The primary etiological factor for the onset and progression of peri-implantitis is the accumulation of a peri-implant plaque biofilm [7,8]. The aim of treatment is to stop the inflammatory processes in the peri-implant tissues and to control local and systemic risk factors that may sustain it [8]. Disruption of the locally accumulating microbial biofilms is a key target [8]. The aim of prevention is to obtain and preserve peri-implant tissues free of clinical inflammation. This is achieved by enabling appropriate self-performed and professionally delivered oral hygiene measures that need to be customized according to the design of implant-supported restorations [8].

Our research group carried out a pilot study in which we observed that although there were no statistically significant differences in the *IL-1A* -889 and *IL-1B* +3954 polymorphisms between the healthy and peri-implantitis groups, there was a trend towards a higher prevalence of genetic polymorphisms in the disease group [52]. The *IL-1RN* (VNTR) polymorphism was not assessed in that study. In the present study, there was no association between the various polymorphisms analyzed (*IL-A* -889, *IL-1B* +3954, and *IL-1RN* (VNTR)) and peri-implantitis. However, the possible association between these genetic polymorphisms and peri-implantitis may be based on alterations in the individual’s immune-inflammatory response. Therefore, the functional impact of these polymorphisms is the appropriate way to study their role in peri-implant disease. Studies that have attempted to associate these polymorphisms only with the occurrence of peri-implantitis are dependent on the multifactorial etiology of the disease [49]. For this reason, especially in cross-sectional studies, it can be difficult to control all the risk factors/indicators and understand the real effect of a particular factor (in this case, genetics) on the development of the disease. 

There are studies that try to relate the existence of certain genetic polymorphisms and the occurrence of peri-implantitis, with contradictory results, and there are also studies that try to assess whether patients with peri-implantitis have higher concentrations of certain inflammatory mediators in the PICF than patients with peri-implant health. However, very few studies have identified genetic determinants, such as polymorphisms, that might be responsible for the overproduction of biochemical mediators (hyperactive phenotype) and, therefore, could be important in determining the risk profile of a patient for peri-implantitis. To the best of our knowledge, only one study has related the presence of polymorphisms in the *IL-1A* and *IL-1B* genes with changes in the levels of the inflammatory mediators IL-1β, plasminogen activator inhibitor type 2 (PAI-2), and PGE2 [35]. In the aforementioned study, when the healthy and peri-implantitis patient groups were combined, subjects who tested negative for IL-1 exhibited higher levels of IL-1β and PGE2 in the PICF than those who tested positive for IL-1 (*p* = 0.02 for IL-1β and *p* = 0.04 for PGE2). In addition, there was a trend towards increased PAI-2 concentrations in IL-1 negative individuals, although the difference was not statistically significant (*p* = 0.052). In our study, we observed that patients with the IL-1 negative composite genotype also had higher concentrations of IL-1β and PGE2, although the difference was not statistically significant (*p* = 0.713 and *p* = 0.634, respectively). For the *IL-1RN* polymorphism (VNTR), it was found that individuals with this polymorphism had higher concentrations of TNF-α, IL-1β, and PGE2 in PICF than individuals with the most common genotype, with statistical significance for TNF-α and PGE2 (*p* = 0.002 and *p* = 0.049, respectively). This may indicate that individuals with the *IL-1RN* polymorphism (VNTR) may have a more vigorous immune-inflammatory response and that although they have not yet developed the disease, they are more likely to have peri-implantitis. This polymorphism was not evaluated in the study by Lachamann et al. [35].

In our study, we also observed that among the IL-1 positive composite genotype individuals, those who had peri-implantitis showed a greater volume of PICF (*p* = 0.042) and a greater concentration of PGE2 (*p* = 0.043). However, in this analysis, the groups had few individuals, and the data must be interpreted with caution. Based on the available evidence, it can be concluded that although some studies do not find a relationship between IL-1β, TNF-α, and PGE2 levels in PICF and peri-implantitis, most of them, as evidenced in systematic reviews and clinical studies, demonstrate a positive association between elevated concentrations of these biomarkers in the PICF of diseased versus healthy sites [20,22,23,32,33,53,54]. The divergent results observed in the aforementioned studies can be attributed to variations in the study design, materials, and methods employed, including differences in sample collection, processing techniques, and assay sensitivity.

Based on our study, there was a correlation between the concentrations of the three mediators evaluated (IL-1β, TNF-α, and PGE2), a fact that was also observed in the study by Lachman et al. for IL-1β, PGE2, and PAI-2 [35]. This fact makes sense, as these inflammatory mediators orchestrate the same biological process. Despite some similarities between studies, differences in some results may be due to the collection methods and biochemical tests used, which differed between studies. The definition of peri-implantitis also varied between studies.

Some limitations of our study should be addressed. The sample size of 51 patients, although a total of 306 analyses were performed, 153 at the biochemical level (IL-1β, TNF-α, and PGE2) and 153 at the genetic level (*IL-1A*, *IL-1B*, and *IL-1RN*), is one of the limitations of the study. However, the only published study that evaluated the relationship between genetic and biochemical aspects in cases of peri-implantitis and peri-implant health included a sample of 29 patients [35]. On the other hand, studies investigating the effect of genetic alterations on the periodontal crevicular immune response have smaller sample sizes [55,56]. Longitudinal studies should be preferred because, although the genetic profile does not vary, biomarkers may not be consistently present during a single instance of PICF collection due to various systemic or local factors [54]. Furthermore, since peri-implantitis has a multifactorial etiology, a study with a longitudinal design would allow better control of the various factors that can contribute to the development of the disease. One of these factors is the potential lack of regular supportive peri-implant care following implant therapy. Follow-up from the planning stage of implant placement would allow better control of the various local risk factors/indicators related not only to the characteristics of the implant, but also to the rehabilitation (the material of the abutment or prosthesis, shape of the abutment), and the surgical site (the amount of keratinized gingiva, the amount of tissue above the neck of the implant). The implants were not all placed at our university, which may make it difficult to analyze various factors that may interfere with the development of peri-implant pathology. Another limitation was the assessment of bone loss using a retroalveolar radiograph (paralleling technique). Due to the 2D nature of this imaging technique, there is an unavoidable overlap of anatomical structures and a lack of 3D information. In addition, it is important to standardize the sampling process for PICF because of the atypical morphology of implant prostheses. The use of different paper strips, cones, membranes, or collection devices can be technique sensitive and potentially lead to inaccurate or misleading results. 

The etiology and pathogenesis of periimplantitis are not currently well understood, and more research is still needed to clarify them. Microbial plaque accumulation around dental implants can trigger inflammatory host response in peri-implant tissues but its progression and severity can vary between individuals. The number of studies that evaluate the role of genetic factors, such as the studied polymorphisms, in the development of peri-implantitis, is quite limited, and some of them do not refer to variables that may impact/influence the results obtained. It would also be important to carry out more studies that try to relate genetic alterations to the consequences at the biochemical level in patients with dental implants. Since peri-implantitis is usually latent in the early stages, the analysis of cytokine levels in the PICF of susceptible individuals can be a valuable analytical tool. Understanding the active components and mechanisms that contribute to this destructive process is crucial in this context. This knowledge has the potential to guide the development of new diagnostic strategies and to identify potential disease markers for peri-implant conditions.

According to the observed results, it is reasonable to suppose that increased levels of TNF-α and PGE2 in the peri-implant tissues would result in more aggressive destruction of both bone and connective tissue in response to the bacterial challenge.

## 4. Materials and Methods

### 4.1. Study Design and Population

The study was conducted in accordance with the ethical guidelines of the Declaration of Helsinki, last revised in 2013. Stringent measures were implemented to ensure the general data protection regulations in place. Approval for this research was granted by the Institutional Review Board (Egas Moniz School of Health and Science, Almada, Portugal) on 24 July 2019 (process number 790).

A total of 27 patients diagnosed with peri-implantitis (bleeding and/or suppuration on probing, probing depth (PD) equal to or greater than 6 mm, bone loss equal to or greater than 3 mm), and 24 individuals with peri-implant health (peri-implant mucosa without inflammatory signs and absence of peri-implant bone loss) were recruited. The diagnostic criteria were in accordance with the updated classification of periodontal and peri-implant diseases [4,57,58].

The convenience sample was obtained from patients referred for periodontal treatment at the Periodontology Department of the Egas Moniz Dental Clinic (EMDC) between July 2021 and July 2022. A screening questionnaire was provided to all potential candidates, and written informed consent was obtained from those whose medical history met the study eligibility criteria and accepted to be enrolled.

Patients were considered eligible if they were born in Portugal, of Caucasian ancestry (i.e., first and second line), unrelated, had a dental implant in function for at least twelve months, were diagnosed with either peri-implant health or peri-implantitis, agreed to participate, and provided informed consent. Patients were excluded if they were pregnant, had an immune disorder with systemic involvement, had recently taken antibiotics (6 months or less), had been on chronic anti-inflammatory therapy (for 6 months or less), had received treatment for the peri-implant condition in the oral region being evaluated, had nonosseointegrated implants, or had a diagnosis of mucositis.

### 4.2. Socio-Demographic and Clinical Variables

Data were collected regarding the following variables: age, sex, smoking status (yes or no), history of periodontitis, and full-mouth plaque score (FMPS). 

Patients suspected of having periodontitis underwent a comprehensive periodontal examination. Peri-implant probing was performed using a CP12 graduated periodontal probe, at six sites around each dental implant, as well as the evaluation of the presence of bleeding or suppuration on probing. Bone loss was assessed using a retroalveolar radiograph (paralleling technique). FMPS was determined by calculating the percentage of total tooth surfaces (four aspects per tooth) that exhibited the presence of plaque. This scoring method was adapted from O’Leary, Drake, and Naylor in 1972 [59].

A single examiner collected all the clinical data in the study (J.M.C.). To ensure consistency and accuracy, a training and calibration exercise was conducted before the study, specifically for PD measurements. The results were statistically analyzed using the intra-class correlation coefficient (intra-examiner reproducibility). The calibration exercise resulted in a high level of agreement, with 95% concordance observed within a range of ±1 mm for the PD measurements.

Plaque control measures were implemented for peri-implant healthy cases, while patients with peri-implantitis were referred for additional treatment in conjunction with reinforcing plaque control measures. If a patient presented multiple implants affected, we included data from the implant with the highest bone loss.

### 4.3. Genetic Analysis

To study genetic polymorphisms, cells were collected from the buccal mucosa, inside of the cheek, using a sterile swab (specifically, Omniswab Whatman^®^ FTA^®^, South Miami, FL, USA, was utilized). They were then transferred to 1.5-mL test tubes (Eppendorf tubes^®^, Hamburg, Germany) and stored at −20 °C for later analysis. The DNA extraction process followed the manufacturer’s protocol as detailed elsewhere (QIAamp^®^ DNA Investigator Handbook, Venlo, The Netherlands).

The polymorphism of the *IL-1RN* gene (rs2234663) analyzed consists of a genetic variation of a repetitive sequence of 86 bp, consisting of a VNTR polymorphism. This sequence can be repeated two, three, four, five, or six consecutive times, providing five possible alleles: 1, 2, 3, 4, and 5. Thus, genotyping using the basic methodology of molecular biology techniques has become the most viable technique. The DNA obtained through the DNA extraction technique was amplified using oligonucleotide sequences specific for this polymorphism (primers). The primers used were: 5′-CTC AGC AAC ACT CCT AT-3′ and 5′-TCC TGG TCT GCA GGT AA-3′. For the *IL-RN* gene polymorphism, the primers were aliquoted at a concentration of 10 μM and the Master Mix was prepared. The Master Mix solution included NZYtaq II 2× Green Master Mix—NZYtech, Lisbon, Portugal (consisting of the Taq DNA Polymerase enzyme, dNTP’s, Buffer, and MgCl2), forward and reverse primers, and RNase/Dnase-free water. The samples obtained with a final volume of 25 μL were inserted into the MJ MINITM Personal Thermal Cycler (Hercules, CA, USA) to carry out the amplification reactions. The initial step involved subjecting the samples to a heating cycle at 96 °C for 1 min. Subsequently, 35 cycles were performed, consisting of denaturation at 94 °C for 1 min, annealing at 60 °C for 1 min, extension at 70 °C for 2 min, and final extension at 70 °C for 5 min. The technique of electrophoresis in 3% agarose gel was used to reveal the genotypes of the polymorphic regions associated with the *IL-1RN* gene (intron 2, VNTR).

For the analysis of the *IL-1A* -889 (rs1800587) and *IL-1B* +3954 (rs1143634) polymorphisms, the real-time polymerase chain reaction (PCR) technique was performed using the commercial kit AppliedBiosystemsTM (Thermo Fisher SCIENTIFIC, Waltham, MA, USA) and using the TaqManTM SNP Genotyping Assay solution (Thermo Fisher SCIENTIFIC, Waltham, MA, USA), which contained the forward and reverse primers and two probes with different fluorescent reporters. This solution is specifically defined for each polymorphism and is commercially available. A Master Mix solution was then prepared containing the TaqManTM Genotyping Master Mix solution (Thermo Fisher SCIENTIFIC, Waltham, MA, USA) and the TaqManTM SNP Genotyping Assay. The reference values were 10 μL for TaqManTM Genotyping Master Mix and 1 μL for TaqManTM SNP Genotyping Assay, which were multiplied by the 51 samples. Subsequently, 11 μL of the previously prepared solution and 9 μL of each DNA sample were added to Eppendorf minitubes. The samples were inserted into the Corbett Research RG 3000 thermocycler (Venlo, The Netherlands) to initiate the amplification reactions in real-time PCR. The results obtained by the Taqman^®^ assay were confirmed by DNA sequencing. Patients with each genotype type were selected for analysis. The results obtained by Sanger sequencing and the TaqMan^®^ assay were identical.

### 4.4. Biochemical Analysis

For biochemical analysis of the inflammatory mediators IL-1β, TNF-α, and PGE2, PICF was collected using paper strips (Periopaper^®^, New York, NY, USA). In cases of peri-implantitis, the paper strips were inserted into the pocket at the most affected site, while in cases of peri-implant health, the collection was performed from the mesio-buccal location. Two collections were made, with an interval of 5 min between them. The analysis was performed one week after the clinical examination. Prior to collection, plaque deposits were removed with a curette, and the collection site was dried and isolated to prevent contamination of the sample with saliva. The procedure involved inserting paper strips into the peri-implant sulcus or pocket until resistance was felt, followed by a 30-s waiting period before removal. Samples that were visually contaminated with blood or saliva were excluded from the analysis. The collected paper strips were then placed in a Periotron^®^ 8000 device (Periotron Ide-Interstate, New York, NY, USA) to measure the volume of PICF collected. Subsequently, the paper strips were carefully transferred to Eppendorf tubes with a filter and stored at −80 °C until they were sent to the Complutense University of Madrid. At the university, the concentrations of the inflammatory mediators IL-1β and TNF-α were analyzed by multiplexed fluorescent sphere immunoassays using the Luminex^®^ 100/200™ system (Austin, TX, USA) and PGE2 by enzyme-linked immunosorbent assay (ELISA). For the analysis of IL-1β and TNF-α, a high-sensitivity kit was used: the MILLIPLEX MAP Human High Sensitivity T-Cell Panel—Immunology Multiplex Assay from Merck Millipore^®^ (Darmstadt, Germany). For PGE2 analysis, a PGE2 ELISA kit from Alpha Diagnostic International^®^ (San Antonio, TX, USA) was used. In the laboratory, samples were processed and analyzed according to the standardized protocols provided by the manufacturers of each of the commercially available kits.

### 4.5. Statistical Analysis

The data were submitted for descriptive and inferential analysis. The association between categorical variables was checked using appropriate inferential statistical methodologies (bivariable analysis/association tests, chi-square (χ^2^), and Fisher’s exact test). Due to the non-normality characteristics of the considered continuous variables, the Mann–Whitney test was used for inferential comparison between groups. For *IL-1A* -889 and *IL-1B* +3954 polymorphisms, the odds ratio (OR) and 95% confidence interval (95%CI) were calculated to assess its association strength with risk of peri-implant disease in two comparison models: dominant (homozygous + heterozygous variant vs. homozygous wildtype) and recessive (homozygous variant vs. homozygous + heterozygous wild- type). The correlation between inflammatory marker concentrations was assessed using Spearman’s rank correlation coefficient. A 5% significance level (*p* ≤ 0.05) was established in the inferential analyses. Data analyses were performed using IBM SPSS Statistics v.28.

## 5. Conclusions

Individuals with the *IL-1RN* gene polymorphism, regardless of peri-implant health status, presented higher levels of TNF-α and PGE2 in PICF compared to patients without the polymorphism, which is believed to place them at a higher risk for peri-implant inflammatory complications.

There are individuals who have not yet developed the disease but may be at greater risk for its development. Since peri-implantitis is a multifactorial disease, it is understood that a single factor may not be sufficient for its development. 

If the association between this polymorphism and the incidence of peri-implantitis is confirmed, carrying out a genetic test will allow the identification of patients susceptible to the disease and the application of rigorous measures to control their risk factors, especially those related to the reduction in inflammation. When there is a history of periodontitis, a similar approach should be implemented.

However, it should be emphasized that in all patients with dental implants, even if they have no risk factors/indicators for the disease, strict plaque control measures are essential for the prevention of peri-implantitis. 

## Figures and Tables

**Table 1:** Genetic polymorphisms have been studied in cases of peri-implantitis.

Genetic Polymorphisms Evaluated in the Studies
IL-1A -889
IL-1A +4845
IL-1B -511
IL-1B +3954
IL-1RN +2018
IL-1RN (VNTR)
IL-6 -174
IL-17 (rs10484879)
IL-17R (rs879576)
TNF-α -308
OPG T950C
OPG G1181C
RANKL (rs9533156)
RANKL (rs2277438)
CD-14 -159

Abbreviations: variable number of tandem repeats (VNTR), tumor necrosis factor-alpha (TNF-α), osteoprotegerin (OPG), receptor activator of nuclear factor-Kappa B ligand (RANKL), cluster of differentiation 14 (CD14).

**Table 2:** Demographic and clinical data of patients with dental implants.

	Peri-Implant Health (*n* = 27)	Peri-Implantitis (*n* = 24)	*p*
Age,			0.865
Mean (SD) (years)	58.74 (8.72)	59.17 (12.40)	
Median (years)	60	57.5	
Range (years)	41–74	38–90	
Sex, *n* (%)			0.121
Male	11 (40.7)	15 (62.5)	
Female	16 (59.3)	9 (37.5)	
Smoking, *n* (%)			0.134
No	18 (66.7)	11 (45.8)	
Yes	9 (33.3)	13 (54.2)	
History of Periodontitis, *n* (%)			0.585
No	11 (40.7)	8 (33.3)	
Yes	16 (59.3)	16 (66.7)	
FMPS (%)			0.234
≤15%	11 (40.7)	6 (25)	
>15%	16 (59.3)	18 (75.0)	
Implant function time			0.960
Mean (SD) (years)	8.6 (4.8)	8.7 (5.8)	
Median (years)	9	6.5	
Range (years)	1–20	1–20	

Abbreviations: Standard deviation (SD), full-mouth plaque score (FMPS).

**Table 3:** Genotype frequencies of *IL-1A* -889, *IL-1B* +3954, and *IL-1RN* (VNTR) polymorphisms, in the peri-implant health group, in the peri-implantitis group, and in the total sample.

Genotype, *n* (%)	Peri-Implant Health (*n* = 27)	Peri-Implantitis (*n* = 24)	Total (*n* = 51)	*p*
*IL-1A* -889				0.714
CC	16 (59.3)	13 (54.2)	29 (56.9)	
CT/TT	11 (40.7)	11 (45.8)	22 (43.1)	
*IL-1A* -889				-
CC	16 (59.3)	13 (54.2)	29 (56.9)	
CT	10 (37.0)	6 (25.0)	16 (31.4)	
TT	1 (3.7)	5 (20.8)	6 (11.7)	
*IL-1B* +3954				0.749
CC	18 (66.7)	17 (70.8)	35 (68.6)	
CT/TT	9 (33.3)	7 (29.2)	16 (31.4)	
*IL-1B* +3954				-
CC	18 (66.7)	17 (70.8)	35 (68.6)	
CT	9 (33.3)	6 (25.0)	15 (29.4)	
TT	0 (0.0)	1 (4.2)	1 (2.0)	
*IL-1RN* (VNTR)				0.811
1/1	23 (85.2)	21 (87.5)	44 (86.3)	
1/5, 2/2	4 (14.8)	3 (12.5)	7 (13.7)	
*IL-1RN* (VNTR)				-
1/1	23 (85.2)	21 (87.5)	44 (86.3)	
1/5	1 (3.7)	0 (0.0)	1 (1.9)	
2/2	3 (11.1)	3 (12.5)	6 (11.8)	

Abbreviations: variable number of tandem repeats (VNTR).

**Table 4:** Analysis of the association between *IL-1A* -889 polymorphism and the risk of peri-implantitis in dominant and recessive genetic models.

Genetic Model *IL-1A* -889	Peri-Implant Health (*n* = 27)		Peri-Implantitis (*n* = 24)		OR (95% CI) *	*p*
	Events	Total	Events	Total		
Dominant TT + CT vs. CC	11	27	11	24	0.81 (0.27–2.47)	0.934
Recessive TT vs. CC + CT	1	27	5	24	0.15 (0.02–1.36)	0.148

Abbreviations: * Odds Ratio (OR) and correspondent 95% Confidence Intervals (95% CI), Mantel-Haenszel.

**Table 5:** Analysis of the association between *IL-1B* +3954 polymorphism and the risk of peri-implantitis in dominant and recessive genetic models.

Genetic Model *IL-1B* +3954	Peri-Implant Health (*n* = 27)		Peri-Implantitis (*n* = 24)		OR (95% CI) *	*p*
	Events	Total	Events	Total		
Dominant TT + CT vs. CC	9	27	7	24	1.21 (0.37–3.99)	0.986
Recessive TT vs. CC + CT	0	27	1	24	-	-

Abbreviations: * Odds Ratio (OR) and correspondent 95% Confidence Intervals (95% CI), Mantel-Haenszel.

**Table 6:** Immunological findings of the total sample in relation to *IL-1A* -889, *IL-1B* +3954, and *IL-1RN* (VNTR) polymorphisms, calculated as relative concentrations (pg/μL of peri-implant crevicular fluid).

Genotype	Number of Individuals	IL-1β (pg/μL) Mean (SD)	TNF-α (pg/μL) Mean (SD)	PGE2 (pg/μL) Mean (SD)
*IL-1A* -889				
CC	29	19.3 (21.9)	1.2 (1.1)	258.5 (332.3)
CT/TT	22	29.0 (59.0)	1.6 (3.5)	177.7 (221.9)
*p*		0.518	0.313	0.231
*IL-1B* +3954				
CC	35	26.1 (48.7)	1.2 (1.3)	262.0 (339.6)
CT/TT	16	18.0 (20.4)	1.6 (3.9)	139.7 (91.3)
*p*		0.776	0.490	0.543
*IL-1RN* (VNTR)				
1/1	44	23.4 (44.2)	0.9 (1.2)	193.8 (265.5)
1/5, 2/2	7	24.1 (24.7)	3.9 (5.5)	411.0 (384.8)
*p*		0.339	**0.002**	**0.049**
IL-1 positive composite Genotype				
Yes	13	18.5 (22.1)	1.8 (4.3)	145.0 (96.9)
No	38	25.2 (46.9)	1.2 (1.3)	250.5 (328.2)
*p*		0.713	0.364	0.634

Abbreviations: Interleukin-1 beta (IL-1β), prostaglandin E2 (PGE2), standard deviation (SD), tumor necrosis factor-alpha (TNFα), variable number of tandem repeats (VNTR), Statistically significant differences (*p* < 0.05) are denoted in bold.

**Table 7:** Immunological findings of patients with peri-implant health and patients with peri-implantitis in relation to the presence of the IL-1 positive composite genotype and *IL-1RN* (VNTR) polymorphism, calculated as relative concentrations (pg/μL of peri-implant crevicular fluid) and relative to peri-implant crevicular fluid volume.

Patients	PICF Mean Volume (μL) (SD)	IL-1β (pg/μL) Mean (SD)	TNF-α (pg/μL) Mean (SD)	PGE2 (pg/μL) Mean (SD)
IL-1 positive composite genotype patients				
Healthy patients (*n* = 8)	0.51 (0.28)	15.31 (18.31)	2.59 (5.53)	119.06 (65.74)
Patients with peri-implantitis (*n* = 5)	0.66 (0.44)	23.65 (28.81)	0.60 (0.50)	186.54 (130.81)
*p*	**0.042**	0.612	0.499	**0.043**
Patients with *IL-1RN* (VNTR) polymorphism				
Healthy patients (*n* = 4)	0.41 (0.56)	31.28 (29.31)	5.56 (7.20)	365.81 (346.89)
Patients with peri-implantitis (*n* = 3)	0.48 (0.29)	14.60 (17.30)	1.68 (0.15)	471.32 (504.10)
*p*	0.660	0.464	0.558	0.464

Abbreviations: Peri-implant crevicular fluid (PICF), Statistically significant differences (*p* < 0.05) are denoted in bold.

**Table 8:** Correlations between concentrations of biochemical markers (IL-1β, TNF-α, and PGE2) (pg/μL).

	Correlation (Rho, Spearman)	
	Rho	*p*
IL-1β vs. TNF-α	0.480	<0.001
IL-1β vs. PGE2	0.382	0.006
TNF-α vs. PGE2	0.528	<0.001

## Data Availability

The data presented in this study are available in the Supplementary Material.

## Supplementary Materials

The following supporting information can be downloaded at: https://www.mdpi.com/article/10.3390/ijms25010651/s1.

## Abbreviations

CD14	cluster of differentiation 14
CI	confidence interval
ELISA	enzyme-linked immunosorbent assay
EMDC	Egas Moniz Dental Clinic
FMPS	full-mouth plaque score
IL-1	interleukin-1
IL-1α	interleukin-1 alpha
IL-1β	interleukin-1 beta
IL-1Ra	interleukin-1 receptor antagonist
OPG	osteoprotegerin
OR	odds ratio
PAI-2	plasminogen activator inhibitor type 2
PCR	polymerase chain reaction
PD	probing depth
PGE2	prostaglandin E2
PICF	peri-implant crevicular fluid
RANKL	receptor activator of nuclear factor-Kappa B ligand
SD	standard deviation
TNF-α	tumor necrosis factor alpha
VNTR	variable number of tandem repeats
